# Determination of Reduced and Oxidized Coenzyme Q_10_ in Canine Plasma and Heart Tissue by HPLC-ECD: Comparison with LC-MS/MS Quantification

**DOI:** 10.3390/antiox8080253

**Published:** 2019-07-30

**Authors:** Anne Marie V. Schou-Pedersen, Dieter Schemeth, Jens Lykkesfeldt

**Affiliations:** 1 Section for Experimental Animal Models, Department of Veterinary and Animal Sciences, University of Copenhagen, Ridebanevej 9, 1870 Frederiksberg C, Denmark; 2 Section for Environmental Chemistry and Physics, Department of Plant and Environmental Sciences, Thorvaldsensvej 40, 1870 Frederiksberg C, Denmark

**Keywords:** coenzyme Q_10_, oxidative stress, congestive heart failure, HPLC, LC-MS/MS

## Abstract

Coenzyme Q_10_ (Q_10_) plays an important role in mammals for energy production in the mitochondria, and as a potent antioxidant. Oxidation ratio (% oxidized in relation to total Q_10_) has been proposed as an important biomarker. A sensitive and reproducible HPLC-ECD method was developed for determination of reduced and oxidized Q_10_ in canine plasma and heart tissue. Chromatographic separation was achieved in 10 min using a Waters Nova-pak C_18_ column and a mobile phase with lithium perchlorate in ethanol/methanol/2-propanol. The validation showed satisfying results. Excellent linear correlation was found (*r*^2^ > 0.9997), intra- and inter-day precisions were below 6.5% (*n* = 5) and recoveries were between 89 and 109% (*n* = 5). Sensitivity stated as Lower Limit of Quantification (LLOQ) was 10 nM. Acceptable stability of both extracted and un-extracted samples was observed. The plasma concentration range of total Q_10_ was found to be between 0.64 and 1.24 µg/mL. Comparison with a developed LC-MS/MS method showed a correlation of *r* = 0.85 for reduced Q_10_ and *r* = 0.60 for oxidized Q_10_ (*N* = 17). However, average results were around 30% lower for ubiquinol using the LC-MS/MS method as compared with the HPLC-ECD analysis. The two methods are therefore not considered to be interchangeable.

## 1. Introduction

Q coenzymes are ubiquitous in eukaryotic cells. Chemically, they consist of a benzoquinone ring coupled to between 6 and 10 isoprenoid units, depending on species [1,2]. Coenzyme Q_10_ (Q_10_) is prevalent in mammals, including dogs, and exists in a reduced and oxidized form, i.e., ubiquinol (CoQ_10_H_2_) and ubiquinone (CoQ_10_) [1,3]. Q_10_ is involved in the electron transport chain, needed to increase the proton gradient in the membrane in order to synthetize ATP and CoQ_10_H_2_ plays an important role as antioxidant preventing lipid peroxidation and oxidative impairment of DNA [4,5]. A shift in oxidation ratio (i.e., % oxidized in relation to total Q_10_) is an indication of increased oxidative stress [3,6]. The heart contains particularly high concentrations of Q_10_ implying that the heart is highly energy-demanding [7]. It could therefore be speculated that the heart is more vulnerable to mitochondrial dysfunction possibly resulting in cardiovascular disease. Several reports have shown a correlation between Q_10_ deficiency and cardiovascular incidents [8,9]. On that basis, Q_10_ has been given as supplement for humans in order to investigate its positive effects on cardiovascular disease. Results from a newer clinical study, where Q_10_ supplement was given as add-on therapy to patients suffering from congestive heart failure, showed a reduction in the relative risk of cardiovascular death of 43% [10].

In order to study the mechanism by which Q_10_ may improve cardiac health in vivo, the use of a relevant comparator model, e.g., the dog, is an obvious option. When assessing the functional status of Q_10_, the total concentration of Q_10_ or oxidation ratio is often determined in plasma. In plasma, Q_10_ is mostly found as CoQ_10_H_2_. However, as the oxidation ratio in plasma is highly affected by dietary uptake and daily rhythm, it may be more relevant to measure the concentration of Q_10_ or oxidation ratio in relevant peripheral tissue [11]. Sample preparation and subsequent analysis is critical for determination of oxidation ratio due to instability of the reduced form of Q_10_ [12,13]. Historically, high-performance liquid chromatography coupled with ultraviolet detection (HPLC-UV) has been used for quantification of the total Q_10_ concentration [7,14]. In order to investigate the oxidation ratio, HPLC coupled to electrochemical detection (HPLC-ECD) has been used extensively, since oxidation ratio may be determined by performing reduction or oxidation in the electrochemical cell after chromatographic separation [13,15,16,17,18]. In recent years, liquid chromatography–mass spectrometry (LC-MS) and liquid chromatography coupled to tandem mass spectrometry (LC-MS/MS) have become important tools in quantitative bio-analysis due to their high sensitivity and selectivity, and several publications have demonstrated the applicability of LC-MS and LC-MS/MS for Q_10_ quantification and evaluation of oxidation ratio in biological samples [19,20,21,22].

In order to pave the road for future experimental and clinical studies using the dog as comparator model of human congestive heart failure, the overall aim of this study was to develop a fast and sensitive HPLC-ECD method for simultaneous quantification of the reduced and oxidized form of Q_10_—i.e., ubiquinol and ubiquinone—in canine plasma and heart tissue. Analysis of heart tissue poses additional challenges as compared to other tissues due to its hard and fibrous nature, and the Q_10_ oxidation ratio in heart tissue has only been scarcely described in the literature [21,23,24,25]. Initially, optimization of sample preparation for heart tissue and plasma was performed, before development of an HPLC-ECD method. Further objectives were: (i) to perform validation of the developed HPLC-ECD method regarding linearity, sensitivity, precision, and accuracy by taking the current FDA bio-analytical guideline into account [26]; and (ii) to compare the HPLC-ECD method with a developed LC-MS/MS method and discuss benefits and drawbacks of using the two detection principles for quantification of total Q_10_ concentration and evaluation of oxidation ratio.

## 2. Materials and Methods

### 2.1. Chemicals and Materials

Ubiquinone (purity > 98%), butylated hydroxytoluene (BHT), 1-propanol, 2-propanol, 96% ethanol, methanol, hexane, lithium perchlorate (LiPerChl·3H_2_0), and ammonium acetate were purchased from Merck (Darmstadt, Germany). Ubiquinol (USP reference standard) was obtained from Sigma Aldrich, now Merck (Darmstadt, Germany). For homogenization of heart tissue, Lysing Matrix A (2 & 4.5 mL) and Lysing Matrix S (2 mL) was purchased from MP Biomedicals (Eschwege, Germany). 5- and 7-mm stainless steel beads were obtained from Qiagen (Manchester, UK). Coenzyme Q_10_-[^2^H_9_] (CoQ_10_-d_9_, purity > 97%) used as internal standard (IS) in the LC-MS/MS analysis was obtained from IsoSciences (Ambler, PA, USA). Stock solutions of CoQ_10_, CoQ_10_H_2_ and CoQ_10_-d_9_ were prepared in hexane at 1000 µM and kept at −80 °C.

### 2.2. Biological Samples

Plasma samples were obtained from client-owned dogs associated with the University of Copenhagen’s surveillance program for myxomatous mitral valve disease [27]. Plasma samples were used for optimization and validation of the HPLC-ECD method and for comparison of the HPLC-ECD and LC-MS/MS methods. All samples were collected following informed consent from the owner and with ethical approval from the Danish Animal Experimentation Inspectorate (Approval no. 2016-15-0201-01074). Canine blood was collected in K_3_-EDTA tubes. Centrifugation at 3000× *g* at 4 °C for 10 min was performed to obtain plasma. Additionally, heart tissue sample was obtained from two dogs from the same program undergoing elective euthanasia. Plasma and heart tissue was processed as described in detail below and stored at −80 °C until analysis.

### 2.3. HPLC-ECD

An Ultimate 3000 HPLC system (Thermo Scientific, Waltham, MA, USA) was used for the HPLC-ECD method. The chromatographic separation was achieved with a Waters Nova-pak C_18_ column with the dimensions 150 × 3.9 mm (4 µm, 60 Å) employing a mobile phase consisting of 20 mM LiPerChl ·3H_2_0 dissolved in ethanol/methanol/2-propanol (75:16.7:8.3, *v*/*v*/*v*). Electrochemical detection was performed with a RS6011 ultra-analytical cell (Thermo Scientific, Waltham, MA, USA) set at 500 mV as determined by a hydrodynamic voltammogram. The guard cell RS6020 was set at −600 mV prior to the analytical cell to reduce all compounds eluting from the column.

### 2.4. LC-MS/MS

LC-MS/MS quantification was performed on a Waters 2795 separation module hyphenated with a Micromass Quattro micro API mass spectrometer with an ESI ionization source (Waters Corp., Milford, MA, USA). The chromatographic conditions from the HPLC-ECD method were used for the LC-MS/MS system with the following adaptations: LiPerChl·3H_2_O was exchanged with 2 mM ammonium acetate and the proportions of the mobile phase components ethanol/methanol/2-propanol were changed to (76:16.9:7.1, *v*/*v*/*v*). The same HPLC column was used, although with an inner diameter of 2.1 mm. The MS system was employed in positive ionization mode using the following parameters (partly adapted from Tang et al. [22]): Capillary voltage: 2.75 kV; cone voltage 30 V; source temperature: 120 °C; and desolvation temperature: 400 °C. Nitrogen was used as desolvation gas (700 L·h^−1^) and cone gas (60 L·h^−1^). Argon was used for collision-induced dissociation. The collision energy was 25 V. Dwell time was 1 s. To exclude salts from entering into the MS, a solvent delay of 4 min was applied. Multiple reaction monitoring (MRM) was used for quantification of CoQ_10_ and CoQ_10_H_2_ as [M + NH_4_]^+^ with the following transitions from precursor to product ions: CoQ_10_: *m*/*z* 881 → 197. CoQ_10_H_2_: *m*/*z* 883 → 197. CoQ_10_-d_9_: 890 → 206. MassLynx software, version 4.1, was used for acquisition and analysis of the data (Waters Corp., Milford, MA, USA).

### 2.5. Optimization of Extraction Procedure

#### 2.5.1. Plasma

Extraction was tested with the solvents 1-propanol, 2-propanol, and hexane:ethanol (50:50, *v*/*v*) in different ratios (2:1, 3:1, 4:1, and 5:1) in relation to plasma.

#### 2.5.2. Heart Tissue

Several factors considered to affect homogenization were tested for their potential influence: Extraction solvents (1-propanol, 2-propanol, hexane:ethanol (50:50, *v*/*v*), beads (5 mm stainless steel beads, 7 mm stainless steel beads, Matrix A and Matrix S), amount of tissue (22 mg, 45 mg, 60 mg, 75 mg and 270 mg), and homogenization time (45 s, 60 s, 120 s). Furthermore, different homogenization methods were tested: Homogenization with a Potter-Elvehjem apparatus (IKA Labortechnik, Staufen, Germany), a FastPrep-24^TM^ (MP Biomedicals, Eschwege, Germany) or mortar and pistil. The effect of freeze-drying and treatment with liquid nitrogen of the heart tissue prior to homogenization was also evaluated. The replicate number was at least three in all optimization experiments.

### 2.6. Final Extraction Procedures

#### 2.6.1. Plasma

75 µL of 1-propanol and 10 µL BHT (10 mg/mL in 96% ethanol) was added to 25 µL of canine plasma kept on dry ice. The solution was mixed for 2 min on a shaker placed at 4 °C followed by centrifugation at 16,000× *g* in 2 min at 4 °C. The supernatant was analyzed immediately. For LC-MS analysis, 2 µL of a 10 µM Coenzyme Q_10_-[^2^H_9_] dissolved in hexane was added before the addition of 1-propanol and BHT.

#### 2.6.2. Heart Tissue

**Figure 1:**
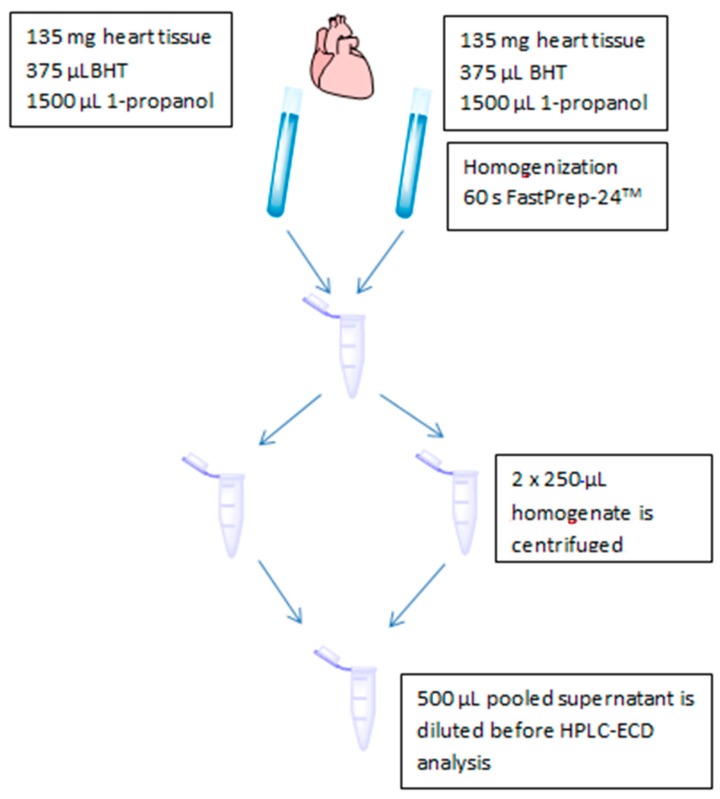
Schematic overview of heart tissue sample preparation before quantification of reduced and oxidized Q_10_ with HPLC-ECD.

The sample preparation for heart tissue is shown in Figure 1. Around 2 × 135 mg of tissue was cut off from a canine heart kept on dry ice; the amounts were weighed exactly and thereafter transferred to two 4.5 mL homogenization tubes containing lysing matrix A, 375 µL BHT (10 mg/mL in 96% ethanol) and 1500 µL 1-propanol. The homogenization tubes were sealed and homogenized for 60 s with an oscillation frequency of 50 Hz on the FastPrep-24. The resulting homogenates were centrifuged using 16,000× *g* at 4 °C for 2 min. Clear supernatants from two homogenizations were pooled and analyzed immediately.

### 2.7. Validation of the HPLC-ECD Method

#### 2.7.1. Calibration Curves and Sensitivity

Before preparation of calibration curves, the 1000 µM solution of CoQ_10_ and CoQ_10_H_2_ prepared in hexane was further diluted to 100 µM with 96% ethanol. The CoQ_10_ stock solution was further diluted 1:1 with 96% ethanol before measurement on a spectrophotometer at 275 nm (ε = 14240). CoQ_10_H_2_ was measured without further dilution at 290 nm (ε = 4010). The spectrophotometric measurements were performed to determine the exact concentrations [28]. Working calibration solutions of CoQ_10_ and CoQ_10_H_2_ at 10 µM in 96% ethanol were used for preparation of the calibration curves. Eight-point calibration curves from 10 to 1000 nM in 96% ethanol were used for both the HPLC-ECD and LC-MS/MS methods. LLOQ was determined as the concentration giving a signal-to-noise ratio (S/N ratio) of 10:1. The precision was investigated at LLOQ by diluting plasma with PBS buffer and homogenate with extraction solution to the expected LLOQ and analyzing the prepared sample five times. Upper Limit of Quantification (ULOQ) was considered to be equal to the highest concentration in the calibration curve at 1000 nM.

#### 2.7.2. Precision and Accuracy

Precision (intra- and inter-assay) and accuracy was estimated in the same set up. 25 µL of canine plasma and around 2 × 135 mg of canine heart tissue were analyzed on three consecutive days (*n* = 5) as is, and with the addition of 25%, 50%, and 75% of CoQ_10_ and CoQ_10_H_2_. Precision was acceptable, if the coefficient of variation, CV%, was within 15%, although a CV% of 20% was acceptable at LLOQ. Accuracy was expressed as % recovery. The requirement for accuracy was a CV% of less than 15%.

#### 2.7.3. Stability

Stability at 4 °C mimicking storage in a cooled auto sampler was investigated for extracted canine plasma and heart tissue every second hour for 8 h. Furthermore, the effect of BHT on the stability of Q_10_ in plasma was evaluated. Extracted samples were tested at −20 °C in up to 4 days. Long-term stability at −80 °C of un-extracted plasma and heart tissue was evaluated for 2 months. A deviation of no more than ± 15% of the nominal concentration was accepted.

#### 2.7.4. Data Analysis

Concentration ranges of CoQ_10_H_2_ and CoQ_10_ are expressed as average ± SD. Regarding precision, the variation was stated as CV%, whereas accuracy was expressed as % recovery of measured concentrations from the expected concentration plus the added concentration of calibration standard. Oxidation ratio was calculated as the concentration of oxidized Q_10_ in relation to the total concentration of Q_10_. Student’s *t*-test was applied for comparison of two groups. Single factor ANOVA was used for comparison of more than two groups. Correlation plots, Bland–Altman plots and statistical calculations were performed in GraphPad Prism 6.0 (GraphPad Prism software, La Jolla, CA, USA). Pearson’s *r* is stated for the correlation plots. Bias ± standard deviation (SD) and 95% limits of agreement are derived from the Bland–Altman plots. A *p*-value of less than 0.05 was considered to be significant.

### 2.8. Comparison of HPLC-ECD with LC-MS/MS

Twenty samples of canine plasma were analyzed with the developed HPLC-ECD method. During the same time frame, comparative data on the 20 samples were collected using the LC-MS/MS method. The integrity of the samples was therefore considered to be the same for both analyses. Data from three dogs were omitted due to an IS response in the LC-MS/MS analysis of almost half of the average response. No additional samples were available for re-analysis. Typical equations for calibration curves obtained using the LC-MS/MS system: ubiquinol: y = 55.4x + 2.12, *r*^2^ = 0.995. Ubiquinone: y = 26.1x + 2.56, *r*^2^ = 0.994. Weighing was not applied.

## 3. Results

### 3.1. Extraction of Q_10_ from Canine Plasma and Heart Tissue

**Figure 2:**
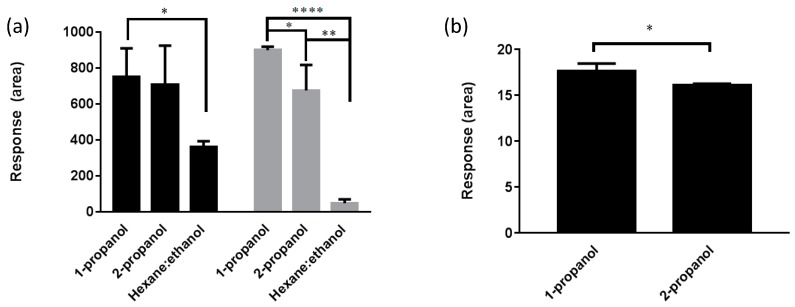
(**a**) Extraction of CoQ_10_H_2_ (_▀_) and CoQ_10_ (**_▀_**) from canine heart tissue (*n* = 3); (**b**) Extraction of CoQ_10_H_2_ (_▀_) from plasma (*n* = 3). * means *p* ≤ 0.05, ** means *p* ≤ 0.01, **** means *p* ≤ 0.0001.

Figure 2a shows results from applying different extraction solvents for extraction of CoQ_10_H_2_ and CoQ_10_ in heart. Heart tissue extraction with 1-propanol resulted in higher and significantly different responses in the HPLC-ECD method as compared to hexane:ethanol (50:50, *v*/*v*) extraction for both CoQ_10_H_2_ and CoQ_10_ (*p* = 0.013 and *p* = 8.4 × 10^−7^, respectively). There was no significant difference between 1-propanol and 2-propanol regarding CoQ_10_H_2._ However, a significant difference was found between 1-propanol and 2-propanol extraction for CoQ_10_ in heart tissue (*p* = 0.045). Furthermore, the variation seemed to be lower with 1-propanol extraction. The homogenization of heart tissue was tested with different procedures; mortar and pistil, Potter-Elvehjem or using a FastPrep-24^TM^. The FastPrep-24^TM^ resulted in fast and efficient homogenization of the heart tissue with CV% of less than 10%, when combined with lysing matrix A in relation to matrix S showing a variation of around 30%. Steel beads measuring 5 mm and 7 mm gave variations between 13 and 24%, respectively. It was possible to downscale the heart tissue amount to 45 mg, thereby reducing extraction volumes equally, since no significant difference was found in a single ANOVA between extraction from the tested tissue amounts (*p* = 0.22). Increasing homogenization time did not result in increased responses (*p* = 0.30). The application of freeze-drying of the heart tissue did not improve the oxidation ratio (*p* = 0.97) as compared to no free-drying. Finally, the application of nitrogen-frozen tissue combined with homogenization by mortar and pistil resulted in low responses for both CoQ_10_H_2_ and CoQ_10_ with unacceptably high variation (CV% > 60%).

Figure 2b shows results from testing different extraction solvents for plasma. A significant difference was found for CoQ_10_H_2_ regarding extraction with 1-propanol and 2-propanol (*p* = 0.038). Plasma extraction of CoQ_10_H_2_ with hexane:ethanol (50/50) was below LLOQ. Extraction of CoQ_10_ was below LLOQ for all solvents in the extraction experiments. Based on the above data, 1-propanol was selected as the best solvent for extraction in both heart tissue and plasma. The lowest possible extraction ratio for plasma was selected to 3:1, which was found to give acceptable responses above LLOQ in the following optimization experiments and a clear supernatant.

### 3.2. Validation of the HPLC-ECD Method

#### 3.2.1. Linearity and Sensitivity

The following equations for calibration curves were typical for CoQ_10_H_2_: y = 0.0590x − 0.3076, *r*^2^ = 0.9998 and for CoQ_10_: y = 0.0139x – 0.0929, *r*^2^ = 0.9997. In plasma and heart tissue, reduced and oxidized Q_10_ demonstrated an LLOQ of 10 nM corresponding to 0.2 pmol on column. The precision at LLOQ for plasma was 2.0% for CoQ_10_H_2_ and 7.2% for CoQ_10_. Regarding heart tissue, the precision at 10 nM was 2.9% for CoQ_10_H_2_, and 4.0% for CoQ_10_. The validated linear range of the method was therefore considered to be from 10 nM to 1000 nM.

#### 3.2.2. Precision and Accuracy 

Precision and accuracy results for plasma and heart tissue can be found in Table 1a,b, respectively. Good reproducibility stated as CV% of less than 6.5% was achieved for both intra-day and inter-day variation for CoQ_10_H_2_ and CoQ_10_. The obtained recoveries in% for accuracy were in a range from 89 to 109% regarding both plasma and heart tissue.

#### 3.2.3. Stability

Stability results can be found in Table 2. Extracted canine plasma and heart tissue were stable for 8 h at 4 °C. Without the addition of BHT, the stability of CoQ_10_H_2_ and CoQ_10_ in plasma was acceptable for 6 h at 4 °C. The addition of BHT is therefore justified. The stability of CoQ_10_H_2_ and CoQ_10_ in plasma and heart tissue was found to be 2 days at −20 °C. CoQ_10_H_2_ and CoQ_10_ were stable in un-extracted canine plasma and heart tissue for at least 2 months at −80 °C.

### 3.3. Comparison of HPLC-ECD with LC-MS/MS

**Figure 3:**
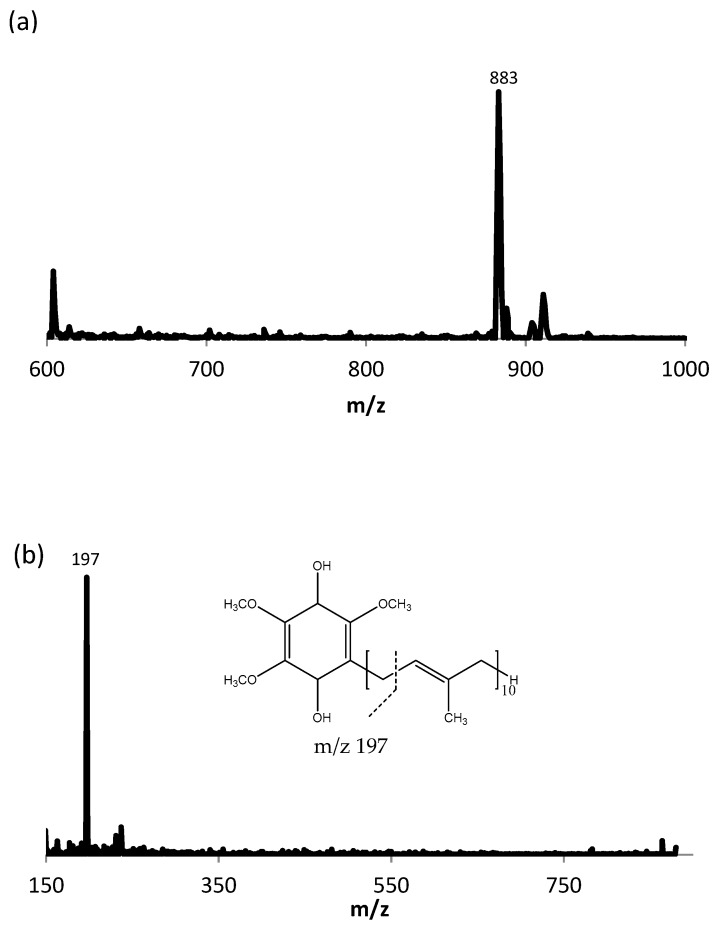
(**a**) Full scan mass spectrum of CoQ_10_H_2_, [M+NH_4_]^+^. (**b**) Product ion mass spectrum of CoQ_10_H_2_ (*m*/*z* of 197)**.**

**Figure 4:**
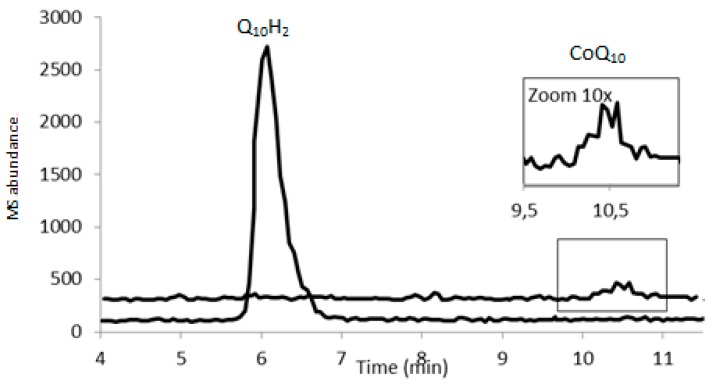
Chromatogram from the MRM trace of a canine plasma sample using the LC-MS/MS method. Lower trace: CoQ_10_H_2_. Upper trace: CoQ_10_. The insert shows 10 times zoom of the CoQ_10_ peak.

**Figure 5:**
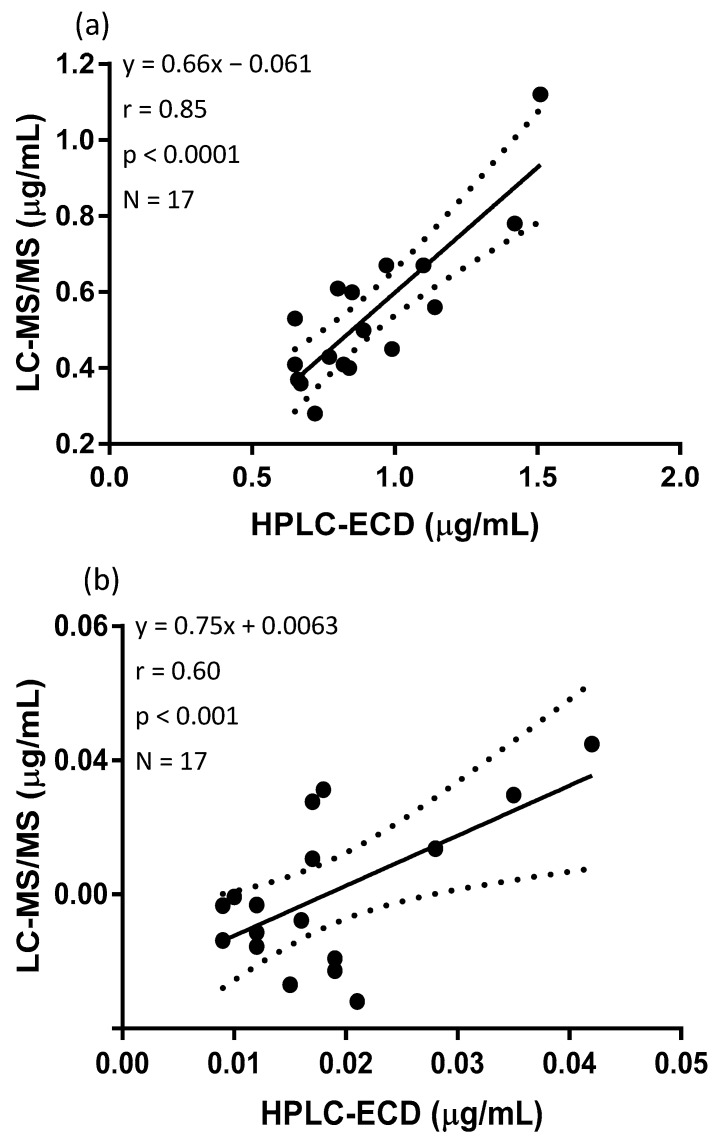
(**a**) Correlation of CoQ_10_H_2_ and (**b**) correlation of CoQ_10_ in canine plasma analyzed by HPLC-ECD and LC-MS/MS. The dotted line corresponds to 95% confidence limits.

**Figure 6:**
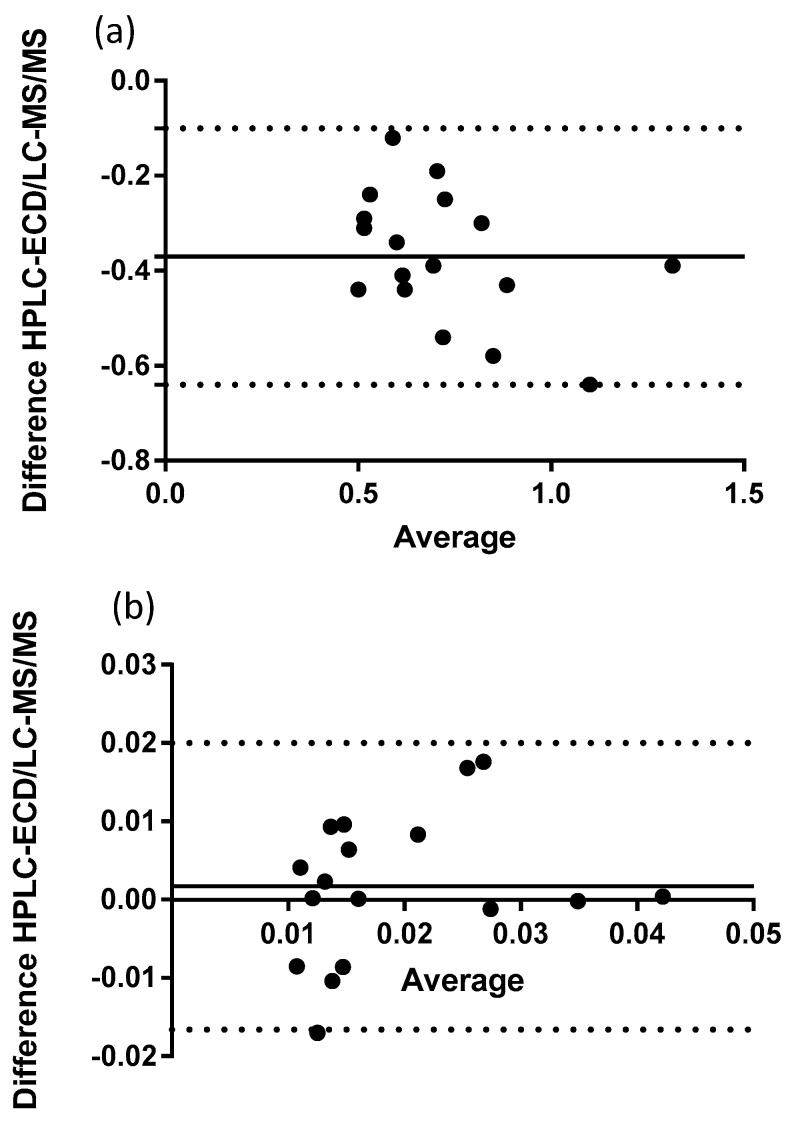
(**a**) Bland–Altman plot of ubiquinol (*N* = 17) and (**b**) Bland–Altman plot of ubiquinone (*N* = 17). The continuous line is the bias. The dotted lines are the 95% limits of agreement.

Seventeen canine plasma samples were analyzed with HPLC-ECD and LC-MS/MS. Both analytical methods were able to quantify the reduced and oxidized forms of Q_10_. Figure 3a shows the full scan mass spectrum of CoQ_10_H_2_ with the product ion mass spectrum of m/z of 197, Figure 3b. The MRM chromatogram traces of CoQ_10_H_2_ and CoQ_10_ are shown in Figure 4. CoQ_10_ was considered to be at the LLOQ of the mass spectrometer. The comparison showed that the correlation for CoQ_10_H_2_ regarding the two methods was acceptable, *r* = 0.85 and for CoQ_10_, *r* = 0.60, see Figure 5a,b, respectively. When including the samples showing low responses of the IS, the correlation for CoQ_10_H_2_ was found to be *r* = 0.73 and for CoQ_10_, *r* = 0.23. Bland–Altman plots are shown in Figure 6. For CoQ_10_H_2,_ Figure 6b, the mean bias ± SD was found to be −0.37 ± 0.14 and the limits of agreement −0.64 to −0.10. The Bland–Altman plot for CoQ_10_, Figure 6b, showed a mean bias ± SD of 0.00172 ± 0.0094 and limits of agreement of −0.017 to 0.02. Both correlation and Bland–Altman plots showed that LC-MS/MS results for ubiquinol were on average around 30% lower as compared to HPLC-ECD results. The intra-day variation of the LC-MS/MS method was estimated from calibration standards to a CV% of 10.8% for CoQ_10_H_2_ and 15.0% for CoQ_10_. The range of total Q_10_ concentration was 0.64 to 1.24 µg/mL for HPLC-ECD in relation to 0.37 to 0.85 µg/mL for LC-MS/MS. The oxidation ratio was determined to 1.9% in the HPLC-ECD analysis and 3.9% using the LC-MS/MS method (*p* = 0.007).

## 4. Discussion

The present study shows optimization and validation of an analytical method for quantification of CoQ_10_H_2_ and CoQ_10_ in canine plasma and heart tissue using HPLC-ECD. As expected, obtaining a homogeneous solution of heart tissue was somewhat challenging. Lysing matrix A containing garnet matrix and a zirconium banded satellite demonstrated the best results in terms of variation. In addition, these materials are considered to have acceptable compatibility with electrochemical detection due to their inert nature. This is in contrast to stainless steel beads that could potentially leak metal ions, and thereby have a negative impact on the S/N ratio of the electrochemical detector or increase post-sampling oxidation. Extraction of tissue amounts between 45 and 270 mg were found to be acceptable. 1-propanol was selected as the best extraction solvent. The usability of 1-propanol for Q_10_ extraction in plasma is in line with previous studies [13,29]. Niklowitz et al. tested extraction of CoQ_10_H_2_ and CoQ_10_ from different swine tissues—including the heart—and found 2-propanol to be the best extraction solvent [23]. However, they did not test 1-propanol. In our study, 1-propanol was significantly better than 2-propanol.

Satisfactory results were obtained during validation of the HPLC-ECD method. Intra-day and inter-day precisions for plasma were comparable with previously published data [22,24,30]. Regarding heart tissue, Pandey et al. [21] measured reduced and oxidized Q_10_ in heart tissue of mice, and found CV% of 17% and 14%, respectively. Tang et al. [24] reported a CV% for heart tissue for both forms of Q_10_ to 20%. Our CV% for reduced and oxidized Q_10_ in heart tissue of less than 6% is therefore markedly better than previously published methods. There was a tendency that recoveries in the accuracy study were below 100% for CoQ_10_H_2_ and above 100% for CoQ_10_. This could be due to ex vivo conversion of the reduced form to the oxidized form of Q_10_ during sample preparation and storage. To maintain the oxidation ratio, it is crucial to keep samples at a low temperature during work up. The stability of extracted samples was acceptable at 4 °C for up to 8 h. Claessens et al. [19] found 1-propanol extracted plasma to be stable in 4.5 h, but no antioxidant was added. The stability of extracted samples was only slightly increased using storage at −20 °C. Furthermore, some of the heart tissue extracted samples, which were clear before freezing, became cloudy after 24 h of freezing. Therefore, it is not advisable to store the Q_10_ extracted samples at −20 °C, and if doing so, at least have three samples available per time point for re-analysis.

Total Q_10_ concentration found in this study with the validated HPLC-ECD method using canine plasma was in a range from 0.65 to 1.24 µg/mL (*n* = 17). In agreement with our data, Svete et al. [31] measured a total coenzyme Q_10_ range in dogs affected by various types and stages of cardiac diseases, and found 25 and 75% percentiles to be between 0.3 and 2.5 µg/mL (*n* = 43) using LC-APCI-MS. A basal Q_10_ range of 0.1 to 0.5 ug/mL (*n* = 8) in healthy dogs was found by Yuan et al. with HPLC-UV detection [14]. Yeramilli-Rao et al. [32] found a basal plasma Q_10_ range in beagle dogs of 0.3 to 0.8 µg/mL (*n* = 8). The detection principle of the latter analysis is unclear. The obtained ranges from HPLC-ECD and LC-MS/MS analysis of canine plasma samples are similar to, or marginally higher than, those reported earlier for dogs. The oxidation ratio for canine plasma and heart tissue determined by the HPLC-ECD method was 1.9% (*N* = 17) and 65% oxidized (*N* = 2), respectively. The observed oxidation ratio in the heart is in good agreement with Niklowitz et al. finding 60% oxidized Q_10_ in swine heart [23]. Thus, although the observed oxidation ratio in the heart can only be regarded as an indication considering the low N-value, it is known that particularly the heart and lung display much higher than average tissue oxidation of antioxidants [33].

The correlation between the HPLC-ECD and LC-MS/MS method was found to be relatively good for both CoQ_10_H_2_ and CoQ_10_. However, actual results for CoQ_10_H_2_ were about 30% lower with the LC-MS/MS method, which is also reflected by the significantly different average oxidation ratio obtained in the two methods. The lower results in the LC-MS/MS method could to some extent be related to differences in calibration, since different calibration stock solutions were used. The variation, especially for CoQ_10_, appeared to be higher in the LC-MS/MS method (between 10.8 and 15.0% for LC-MS/MS in relation to less than 6.5% for HPLC-ECD). This could partly be explained by the fact that measurement of CoQ_10_ was near or at the LLOQ of the LC-MS/MS instrument making especially the CoQ_10_ comparison uncertain. Furthermore, the addition of a small volume of IS in hexane (2 µL) could have contributed to the increased variation observed in the LC-MS/MS method. The oxidation ratio obtained for plasma samples using LC-MS/MS was higher than for HPLC-ECD, which could indicate increased oxidation of CoQ_10_H_2_. The auto sampler connected to the MS instrument could only run with a temperature of around 8 °C, which may not have been sufficient to ensure stability of the analytes. Another explanation could be that oxidation of CoQ_10_H_2_ took place in the emitter, thereby increasing the concentration of CoQ_10_. However, the chromatographic trace from CoQ_10_ at the retention time of CoQ_10_H_2_ showed no visible peak for CoQ_10_ In conclusion, the data set used for comparing the two methods is quite small, making the method comparison preliminary and further investigations regarding the observed discrepancy is warranted.

The sensitivity of the HPLC-ECD method of 10 nM with an S/N ratio of 10 was found to be similar to or better than previous methods for Q_10_ quantification. An LOD based on a S/N ratio of 3 has been reported to 17 nM using HPLC-ECD [13]. Finckh et al. [15] found LOQs between 4 and 12 nM in plasma with an S/N ratio of 5. An LOQ of 17 nM for CoQ_10_H_2_ and 6 nM for CoQ_10_ was reported using UPLC-MS/MS [22]. The sensitivity of the developed LC-MS/MS method was comparable with the HPLC-ECD method regarding CoQ_10_H_2_, but, quite surprisingly, actually poorer regarding analysis of CoQ_10_. The utilization of 100% organic solvent in the mobile phase could potentially have led to lower responses, and poorer sensitivity of the mass spectrometer due to loss of protonation in the gas phase to the organic solvents as described in [34]. Only optimization of instrument parameters were performed in the MS analysis; the same column and mobile phase were used in both methods. Improved sensitivity could perhaps have been obtained by running the samples with negative APCI LC-MS as described by Hansen et al. [20].

## 5. Conclusions

A simple and rapid HPLC-ECD method with a good precision (<6.5%), accuracy (89–109%), and sensitivity (LLOQ of 10 nM) was developed for the quantification of CoQ_10_H_2_ and CoQ_10_ in canine plasma and heart tissue. Furthermore, the stability of CoQ_10_H_2_ and CoQ_10_ during sample preparation, analysis and storage was found to be acceptable. Our results indicate a difference between the results obtained by HPLC-ECD and LC-MS/MS, perhaps due to differences in calibration stock solutions, or increased oxidation during storage or analysis in the LC-MS/MS system. The two methods are therefore not considered to be interchangeable. The observed discrepancy could be investigated further by analyzing extracted heart tissue samples with the two developed methods.

## Figures and Tables

**Table 1:** (a) Precision (CV%) and accuracy (recovery%) for the quantification of CoQ_10_H_2_ and CoQ_10_ in canine plasma determined within one day (*n* = 5) or in three different days (*n* = 5). (b) Precision (CV%) and accuracy (recovery%) for the quantification of CoQ_10_H_2_ and CoQ_10_ in canine heart tissue determined within one day (*n* = 5) or in three different days (*n* = 5).

**(a)**					
**Analyte**	**Concentration Added (nM)**	**Intra-Day**		**Inter-Day ^1^**	
		**CV%**	**Recovery%**	**CV%**	**Recovery%**
**CoQ_10_H_2_**	40	1.73	91.9	1.93	95.0
	80	6.00	98.8	3.53	93.2
	119	0.89	94.2	1.37	89.1
**CoQ_10_**	11	3.46	90.9	6.49	88.8
	22	2.89	88.9	4.42	101
	33	2.06	86.7	4.24	109
**(b)**					
**Analyte**	**Concentration Added (nM)**	**Intra-Day**		**Inter-Day ^1^**	
		**CV%**	**Recovery%**	**CV%**	**Recovery%**
**CoQ_10_H_2_**	890	5.48	89.6	3.75	90.7
	1775	2.50	96.4	3.43	91.5
	2660	3.96	94.1	2.49	94.0
**CoQ_10_**	7516	4.06	109	2.90	107
	15,033	4.47	92.1	3.08	106
	22,549	5.02	107	3.70	103

^1^ Average of three different days (*n* = 15).

**Table 2:** Stability results of CoQ_10_H_2_ and CoQ_10_ in extracted canine plasma and heart tissue at 4 °C and −20 °C and un-extracted canine plasma and heart tissue at −80 °C (*n* = 3). Extracted canine plasma was investigated with and without the addition of the antioxidant BHT at 4 °C.

Analyte (%)	Autosampler (4 °C, 8 H) w/BHT		Autosampler (4 °C, 6 H) w/o BHT	Short-Term (−20 °C, 2 d)		Long-Term (−80 °C, 2 m)	
	Plasma	Heart	Plasma	Plasma	Heart	Plasma	Heart
**CoQ_10_H_2_**	91	103	86	89.9	94.8	108	107
**CoQ_10_**	114	115	115	112	119	87	106
